# METTL3 promotes drug resistance to oxaliplatin in gastric cancer cells through DNA repair pathway

**DOI:** 10.3389/fphar.2023.1257410

**Published:** 2023-09-26

**Authors:** Yi Wang, Zhongshi Hong, Jintian Song, Peilin Zhong, Liang Lin

**Affiliations:** ^1^ Department of Gastrointestinal Surgical Oncology, Fujian Cancer Hospital, Clinical Oncology School of Fujian Medical University, Fuzhou, China; ^2^ Department of General Surgery, The Second Affiliated Hospital of Fujian Medical University, Quanzhou, China; ^3^ Department of Abdominal Oncology, Fujian Cancer Hospital, Clinical Oncology School of Fujian Medical University, Fuzhou, China; ^4^ Department of Gynecology, Fujian Cancer Hospital, Clinical Oncology School of Fujian Medical University, Fuzhou, China

**Keywords:** gastric cancer (GC), oxaliplatin (OXA), methyltransferase-like 3 (METTL3), DNA repair, drug resistance

## Abstract

Gastric cancer (GC) poses a significant threat to human health and remains a prevalent form of cancer. Despite clinical treatments, the prognosis for Gastric cancer patients is still unsatisfactory, largely due to the development of multidrug resistance. Oxaliplatin (OXA), a second-generation platinum drug, is commonly recommended for adjuvant and palliative chemotherapy in Gastric cancer; however, the underlying mechanisms of acquired resistance to Oxaliplatin in Gastric cancer patients are not yet fully understood. In this study, we aimed to explore the potential mechanisms of Oxaliplatin resistance in Gastric cancer by employing bioinformatics analysis and conducting *in vitro* experiments. Specifically, we focused on investigating the role of methyltransferase-like 3 (METTL3). Our findings revealed that the knockdown of METTL3 significantly impeded the proliferation and migration of Gastric cancer cells. METTL3 knockdown induced apoptosis in OXA-resistant Gastric cancer cells and enhanced their sensitivity to Oxaliplatin. Furthermore, we found that DNA repair pathways were significantly activated in OXA-resistant Gastric cancer cells, and METTL3 knockdown significantly inhibited DNA repair pathways. Another important finding is that METTL3 knockdown and OXA-induced Gastric cancer cell death are additive, and the targeted METTL3 can assist Oxaliplatin treatment. Collectively, our findings suggest that METTL3 knockdown can augment the sensitivity of Gastric cancer cells to Oxaliplatin by impeding DNA repair processes. Consequently, targeting METTL3 holds great promise as a viable adjuvant strategy in the treatment of Gastric cancer patients.

## Introduction

Stomach cancer, also referred to as gastric cancer (GC), is a prevalent malignancy originating from the cells that line the stomach (Venerito et al., 2016; Smyth et al., 2020). It represents a common form of cancer and remains a substantial cause of cancer-related fatalities worldwide. The role of m6A modification in stomach cancer has emerged as a promising research area, with studies shedding light on its potential implications in the development and progression of this disease (Wang et al., 2020). Multiple studies have reported discernible alterations in m6A modification patterns within stomach cancer tissues in comparison to normal gastric tissues (Zhang et al., 2020; Meijing et al., 2022). These observed changes in m6A levels and distribution suggest a plausible involvement in the process of gastric carcinogenesis. Dysregulation of m6A regulatory proteins has been noted in stomach cancer, exemplified by the upregulation of Methyltransferase-like 3 (METTL3), a critical m6A “writer,” in gastric cancer tissues, which has been associated with an unfavorable prognosis (Wei et al., 2022). METTL3-mediated modification of N6-methyladenosine was found to be required for the process of epithelial mesenchymal transformation and metastasis in GC (Yue et al., 2019). METTL3 serves as the primary catalytic enzyme within the RNA methylation system, typically forming a stable core heterodimeric complex alongside METTL14 (Fu et al., 2014; Liu et al., 2014). Moreover, an ensemble of co-factors, including Wilms tumor 1-associating protein (WTAP), Vir-like m6A methyltransferase-associated (VIRMA) protein, RNA-binding motif protein 15/15B (RBM15/15B), and zinc finger CCCH-type containing 13 (ZC3H13) protein, collectively contribute to the activity and specificity of METTL3-associated m6A regulation (Fu et al., 2014; Ping et al., 2014; Knuckles et al., 2018). Dysregulated METTL3 has shown promise as a potential prognostic marker for GC. In the treatment of GC, drug resistance poses a significant challenge, representing the ability of cancer cells to withstand the impact of anticancer drugs, ultimately leading to reduced treatment effectiveness and disease progression.

METTL3, an RNA methyltransferase and a crucial component of the m6A mRNA modification machinery, plays a pivotal role in regulating gene expression through m6A modification (Song and Zhou, 2021). However, emerging evidence suggests that METTL3’s involvement extends beyond gene regulation and encompasses drug resistance in various cancer types, including GC. Numerous studies have reported elevated levels of METTL3 expression in GC tissues, and this upregulation has been consistently linked to chemoresistance. Increased METTL3 expression has been directly associated with a poor response to chemotherapy and diminished survival rates among patients. For instance, METTL3 has been found to bolster chemoresistance by upregulating the expression of anti-apoptotic genes, such as Bcl-2 and Bcl-xL, as well as drug efflux transporters like ABCB1 (P-glycoprotein) (Pan et al., 2021; Li H. et al., 2022; Ouyang et al., 2023). Given these findings, METTL3 emerges as a potential target for combating drug resistance in GC, warranting further research and investigation.

**FIGURE 1:**
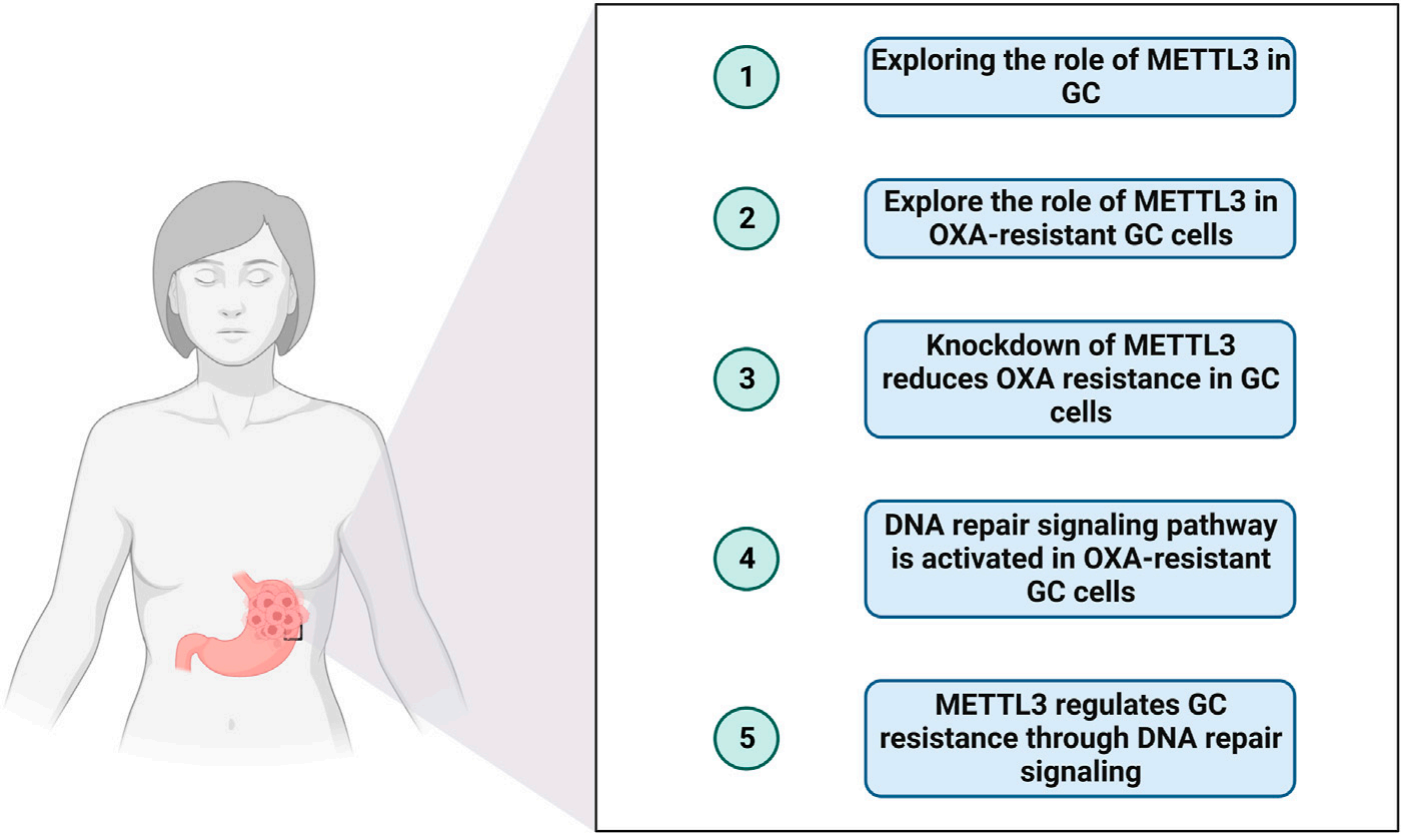
Flowchart.

Oxaliplatin (OXA), an antineoplastic medication, is utilized in the treatment of various cancer types (Bang et al., 2012). Its mechanism of action involves interfering with the replication process of cancer cells, leading to their subsequent demise. OXA, when combined with other chemotherapy agents such as fluorouracil (5-FU) and leucovorin, has been extensively researched and employed in the management of advanced gastric cancer (Cavanna et al., 2006; Dos Santos et al., 2022). Typically administered intravenously, these combination regimens are designed to target cancer cells throughout the body, thereby impeding disease progression and enhancing survival rates. Nevertheless, the emergence of OXA resistance remains an inevitable challenge during gastric cancer treatment, significantly impacting patient management. In this study, we investigate the potential mechanism underlying OXA resistance centered around METTL3. We focused on the DNA repair pathway in OXA-resistant GC cells and performed functional experiments on OXA-resistant GC cells to explore the potential role of METTL3. The research idea of this study is shown in Figure 1.

## Methods

### Cell culture

All mammalian cell lines used in this study were grown in a humidified 37 °C incubator with 5% CO_2_. Human gastric epithelial cell line GES1 and gastric cancer cell lines AGS and HGC27 were purchases from Shanghai Institute of Biochemistry and Cell Biology, Chinese Academy of Sciences (Shanghai, China). OXA-resistant gastric cancer cell lines, AGS-R and HGC27-R, were generated by subjecting cells to increasing concentrations of OXA (Ren et al., 2023). The process involved gradually exposing the cells to OXA at ascending concentrations ranging from 0.25 μg/mL to 6 μg/mL and 2 μg/mL, respectively. DNA repair inhibitors, namely, VE-821 (10 μM) and KU-55955 (10 μM), were procured from Selleckchem. Unless explicitly mentioned, the duration of drug treatment was set at 24 h.

### Extraction of RNA and quantitative real-time PCR (qRT-PCR)

Total RNA was extracted from cells using TRIzol reagent (Invitrogen) following the manufacturer’s instructions. The concentration of total RNA was determined by measuring the absorbance at 260 nm using an Agilent 2100 Bioanalyzer. For quantitative real-time polymerase chain reaction (qRT-PCR), an ABI Illumina instrument (Foster, United States of America) was utilized, and SYBR Green (Tiangen) was used as the fluorescent dye. Use NTC (No Template Control) wells to detect contamination or non-specific amplification. PCR conditions were set: 95 °C for 30 s, followed by 40 cycles alternating between 95 °C for 5 s and 60 °C for 30 s. Each experiment was repeated at least three times, and the relative mRNA expression levels were calculated using the 2^−ΔΔCT^ method. The primer sequences used were shown in Table 1.

**TABLE 1:** The primer sequences.

Gene	5′→3′Forward primer	5′→3′Reverse primer
XCCR1	GGA​GGA​CCT​CAC​TGA​GAT​CAG​G	GGG​CTG​GCA​CAG​TGA​CTT​CAC
RAD51	ATC​CCT​GCA​TGC​TTG​TTC​TC	CTG​CAG​CTG​ACC​ATA​ACG​AA
PARPL	AAG​TGC​CAG​TGT​CAA​GGA​GA	ACA​GGG​AGC​AAA​AGG​GAA​GA
METTL	CAA​GCT​GCA​CTT​CAG​ACG​AA	GCTTGGCGTGTGGTCTTT
GAPDH	GGA​CCT​GAC​CTG​CCG​TCT​AG	GTA​GCC​CAG​GAT​GCC​CTT​GA
Β-ACTIN	CCA​GAT​CAT​GTT​TGA​GAC​CTT​CAA	GTG​GTA​CGA​CCA​GAG​GCA​TAC​A

### Establishment of overexpression or knock-down GC cell lines

Gene silencing was accomplished through the application of gene-specific small interfering RNAs (siRNAs), which were procured from Hanbio Biotechnology Co., Ltd. (Shanghai, China). The siRNAs used in the study are listed in Supplementary Table S1. The transfection of siRNAs was performed via a technique known as “reverse transfection.” To prepare the transfection mix, 10 nM (final concentration) of siRNA, along with Lipofectamine RNAiMAX and OPTI-MEM (both from Invitrogen, United States of America), were combined following the instructions provided in the RNAiMAX manual. For the purpose of METTL3 overexpression, an expression plasmid (GV144, synthesized by GeneChem, Shanghai) was employed, while an empty vector was utilized as the negative control (NC).

### RNA m6A quantification analysis

M6A levels were evaluated using a colorimetric assay employing the RNA m6A quantification kit (ab185912, Abcam, United States of America). A sample size of 200 ng RNA was utilized, and the measurements were recorded at 450 nm. The obtained results were normalized in relation to the control samples (GSE1).

### Cell viability and wound healing

Cell viability was detected by Cell Counting Kit-8 (Beyotime Institute of Biotechnology, Jiangsu, China). Cells (3 × 10^3 cells/well) were incubated with 10 μL Counting Kit-8 reagent in a 96-well plate, and then the absorbance was measured at a wavelength of 450 nm. 5 × 10^5 cells (per well) are seeded into 6-well culture plates and incubated until 80% confluence is reached. Then, a sterile 200ul pipette tip is used to score the center of the cell layer. 48 h later, photographs are taken to estimate wound healing. The details of the method can be found in the previously published article (Guo et al., 2017; Ma et al., 2020; Wang C. et al., 2021).

### Western blotting

The Western blotting (WB) technique was executed following standardized protocols. Total cellular or tissue protein was lysed using RIPA buffer supplemented with protease inhibitor and phosphatase inhibitor cocktail. The protein content was quantified using the BCA Protein Assay Kit (Thermo Fisher Scientific, Catalog number: J63283.QA). The antibodies employed in this study included Anti-METTL3 (1:1,000 dilution; cat. no. 15073-1-AP; ProteinTech Group, Inc.), β-Actin (23,660–1-AP, Proteintech, Wuhan, China), GAPDH (1:1,000 dilution; product no. D16H11; Cell Signaling Technology, Inc.), and anti-p-γH2AX (1:1,000 dilution; product no. 9718S; Cell Signaling Technology, Inc.). The secondary antibody was procured from ABclonal Biotech Co., Ltd. The specific methodology followed in this study is described in previous articles (Kurien and Scofield, 2006; Mishra et al., 2017; Walentowicz-Sadlecka et al., 2019; Li et al., 2021).

### Bioinformatics analysis

Publicly available data for gastric cancer were retrieved from the TCGA database (Qing et al., 2022; Yuan et al., 2022). Specifically, mRNA expression profile data and corresponding clinical information for 348 gastric cancer patients and 31 controls were obtained from the TCGA-STAD cohort database. To predict the prognosis of gastric cancer patients in relation to the mRNA expression level of METTL3, we utilized the Kaplan-Meier Plotter online analysis website (Nagy et al., 2021). Additionally, the expression levels and clinical relevance of METTL3 were examined using the UALCAN database (Chandrashekar et al., 2022). Furthermore, the correlation between genes was visualized using the TIMER web server (Li et al., 2017).

### Statistical analysis

Statistical analysis was performed using R version 4.1.2, and GraphPad Prism 8.0 (Wang Q. et al., 2021; Wu et al., 2021; Fang et al., 2022; Liu et al., 2022). Each experiment was repeated independently a minimum of three times. The Student’s t-test was utilized to determine significant differences between groups for continuous variables. Post hoc comparisons for variables that exhibited differences among multiple groups were conducted using Tukey’s test. A significance level of *p* < 0.05 was considered to indicate statistically significant differences.

## Results

### METTL3 exhibits overexpression in gastric cancer and is correlated with a poor prognosis

**FIGURE 2:**
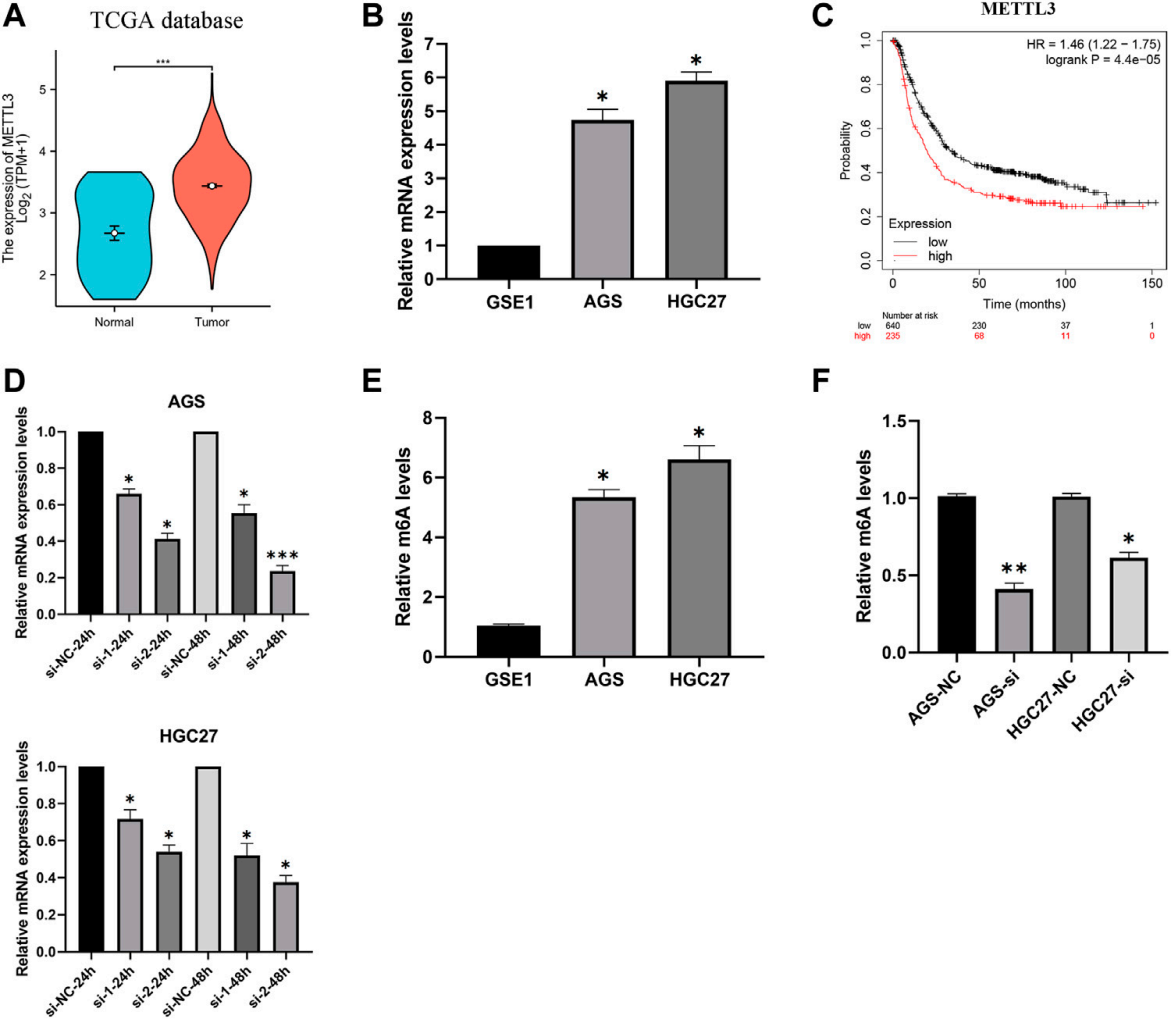
METTL3 is overexpressed and associated with the prognosis of GC patients. **(A)** METTL3 was significantly overexpressed in GC patients. **(B)** METTL3 was significantly highly expressed in GC cells. **(C)** High expression of METTL3 is associated with poor prognosis in GC patients. **(D)** Knockdown of METTL3 in GC cell lines. **(E)** m6A methylation levels in GC cell lines. **(F)** m6A methylation levels in GC cell lines were significantly reduced after METTL3 knockdown. *, *p* < 0.05; **, *p* < 0.01; and ***, *p* < 0.001.

Analysis of gastric cancer patients (TCGA-STAD) in the TCGA database revealed higher expression levels of METTL3 in tumor samples (Figure 2A). Furthermore, our investigation of mRNA expression levels in GC cells (AGS and HGC27) demonstrated significant overexpression of METTL3 compared to normal cells (Figure 2B). This overexpression of METTL3 was associated with an unfavorable prognosis in GC patients (Figure 2C). To delve deeper into the potential biological role of METTL3 in GC, we employed METTL3 knockdown in GC cells. Among the siRNAs tested, si-2 demonstrated a more effective knockdown of METTL3 (Figure 2D). si-2 was used in subsequent knockdown experiments. Aberrant m6A methylation levels were observed in gastric cancer cells, and upon knocking down METTL3, the m6A methylation levels in gastric cancer cells were significantly reduced (Figure 2E; 2F). These findings highlight the relevance of m6A methylation in gastric cancer and suggest a key role for METTL3 in regulating this process.

### Relationship between METTL3 and clinical characteristics of GC patients

**FIGURE 3:**
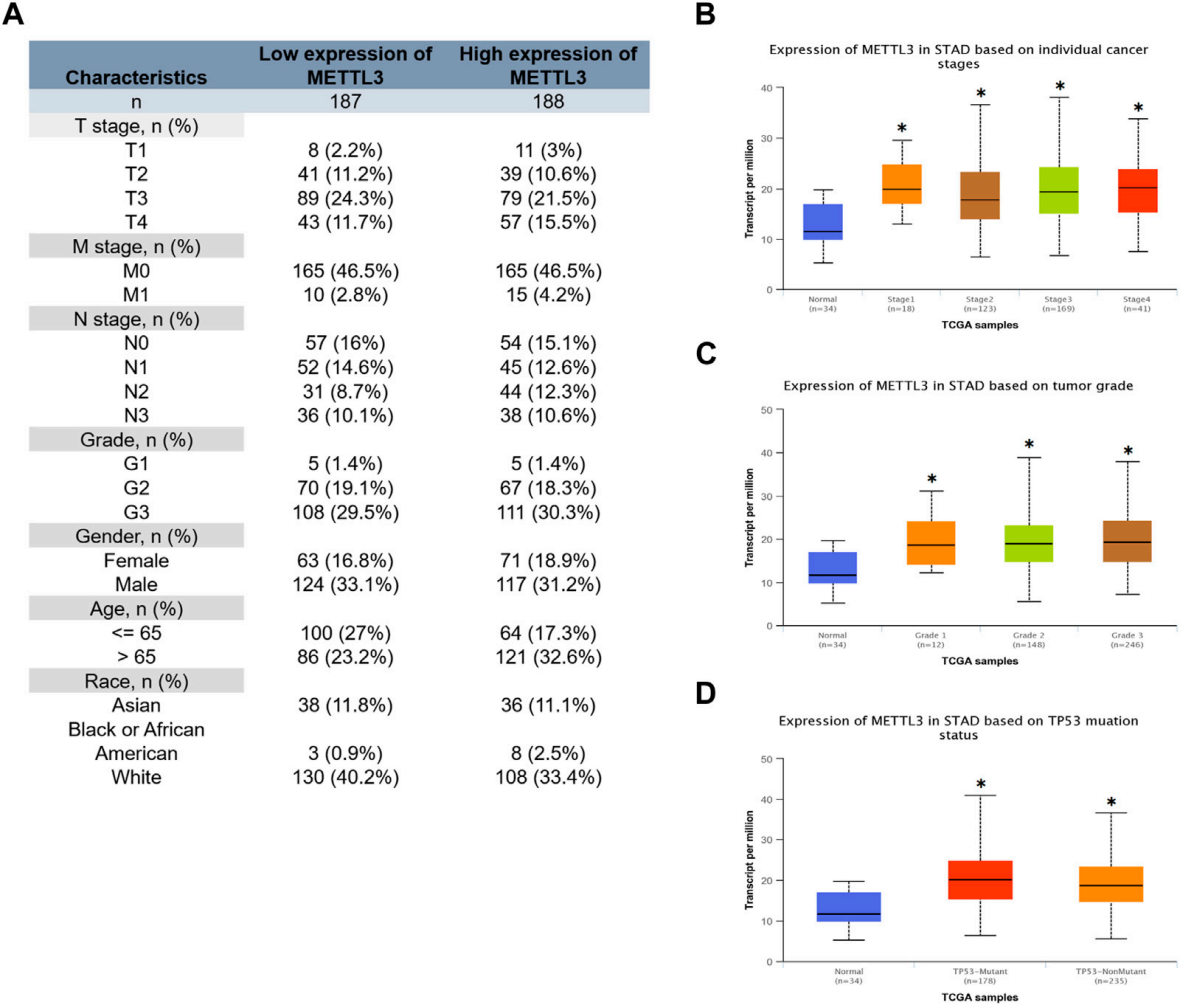
Relationship between METTL3 and clinical characteristics of GC patients. **(A)** Clinical information for the high and low METTL3 groups. Analysis of the relationship between METTL3 and clinical characteristics. **(B)** Stage. **(C)** Grade. **(D)** TP53 mutation status. *, *p* < 0.05; **, *p* < 0.01; and ***, *p* < 0.001.

The clinical characteristics data of GC patients were retrieved from the TCGA database. Patients were stratified based on the median value of mRNA expression levels of METTL3. The specific clinical information is illustrated in Figure 3A. Our analysis revealed that the high METTL3 group exhibited a greater proportion of patients with T4 and M1 stages (Figure 3A). Furthermore, we conducted additional investigations and observed that the cancer group displayed higher levels of METTL3 expression compared to the normal group (Figures 3B–D). However, no statistically significant differences were observed among the various cancer stages (Figure 3B) and grades (Figure 3C) in terms of METTL3 expression. It is noteworthy that cancer patients with a TP53 mutant phenotype exhibited elevated levels of METTL3 expression (Figure 3D).

### METTL3 exhibits an association with the invasion and migration of GC cells

**FIGURE 4:**
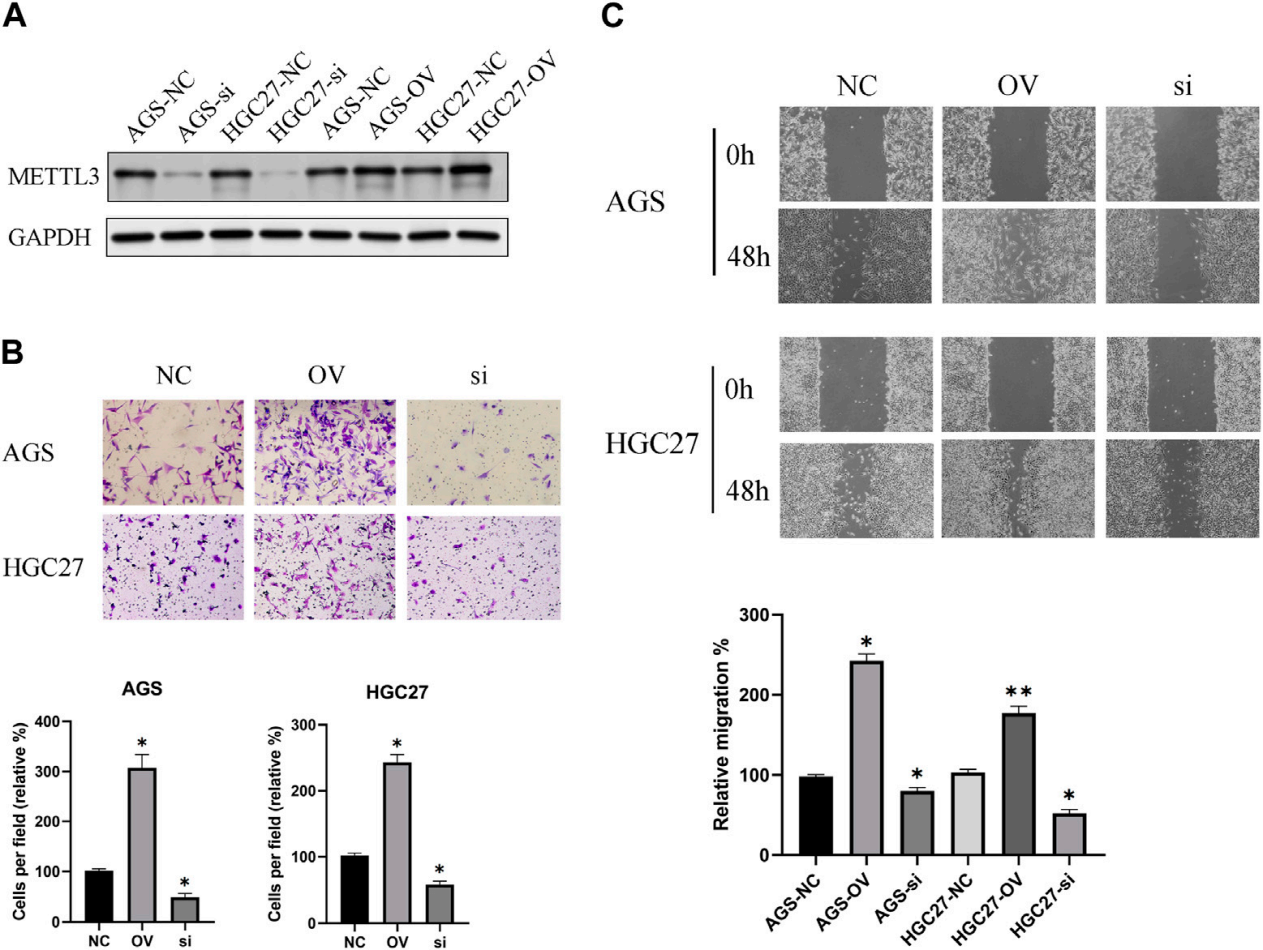
METTL3 exhibits an association with the invasion and migration of GC cells. **(A)** Examination of METTL3 knockdown and overexpression at the protein level. **(B)** The relationship between METTL3 and GC cell invasion. The image shows the results taken under a 10x microscope. **(C)** The relationship between METTL3 and GC cell migration. The image shows the results taken under a 4x microscope. *, *p* < 0.05; **, *p* < 0.01; and ***, *p* < 0.001.

The effectiveness of METTL3 knockdown and overexpression was corroborated through protein blotting analysis (Figure 4A). Subsequently, leveraging the successful modulation of METTL3 expression, we conducted an in-depth exploration of its involvement in the processes of GC cell invasion and migration. Notably, our investigations revealed a substantial promotion of GC cell invasion upon METTL3 overexpression, leading to a notable 2–3 fold increase in invasion capacity. Conversely, the knockdown of METTL3 significantly impeded GC cell invasion, resulting in a 50% reduction in invasive capacity (Figure 4B). Similarly, our migration assay on GC cells yielded analogous outcomes (Figure 4C). These findings strongly indicate the regulatory role of METTL3 in the invasion and migration processes of GC cells. High expression of METTL3 may be a phenotypic marker of GC malignancy.

### METTL3 associated with drug resistance in OXA-resistant GC cells

**FIGURE 5:**
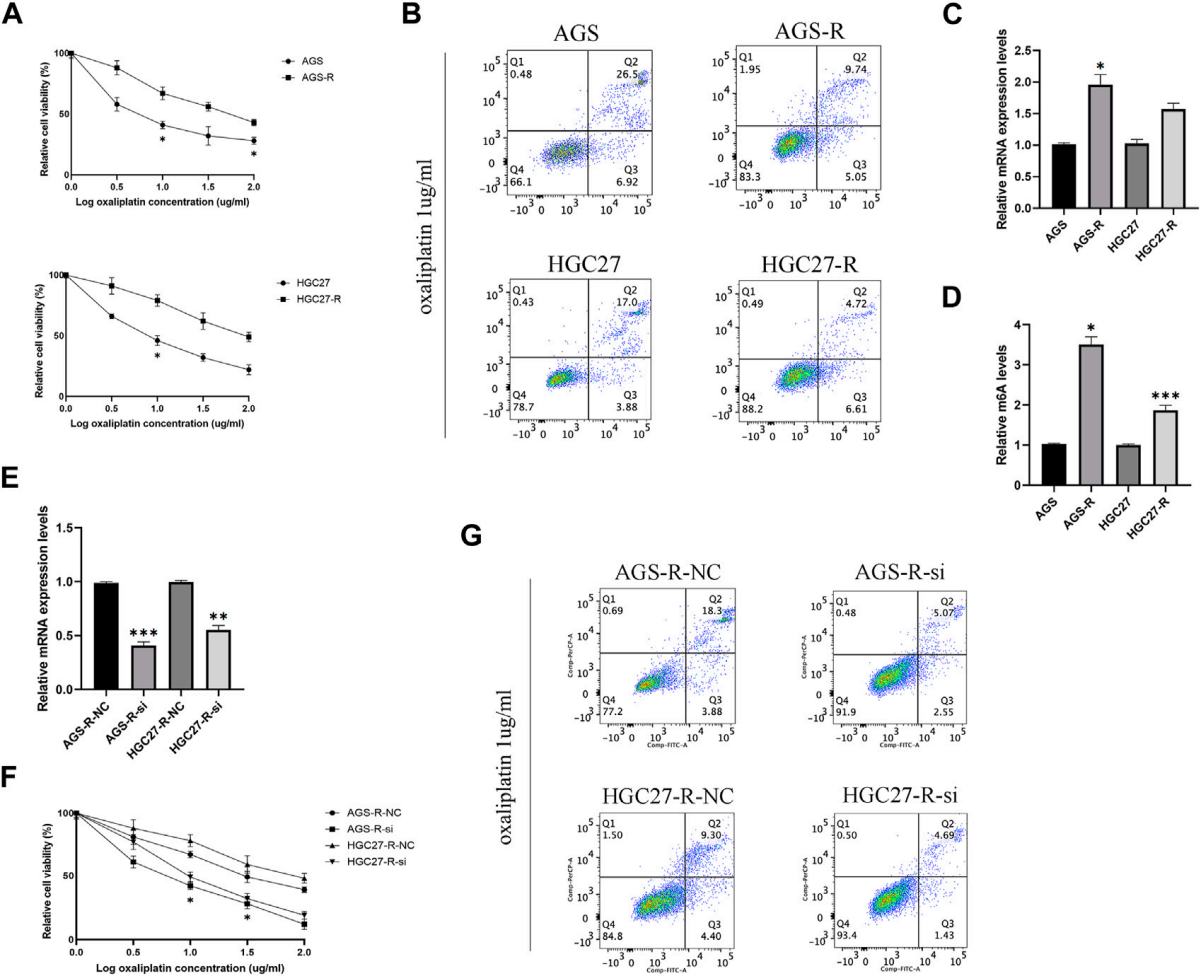
METTL3 associated with drug resistance in OXA-resistant GC cells. **(A)** The cell viability was compared between parental and OXA-resistant GC cells. **(B)** Apoptosis assay of parental and OXA-resistant GC cells after OXA treatment. **(C)** Expression levels of METTL3 in parental and OXA-resistant GC cells. **(D)** m6A levels of METTL3 in parental and OXA-resistant GC cells. **(E)** Relative mRNA expression levels in OXA-resistant GC cells after METTL3 knockdown. **(F)** OXA-resistant GC cells exhibit decreased cell viability after METTL3 knockdown. **(G)** METTL3 knockdown reduces drug resistance in OXA-resistant cells. *, *p* < 0.05; **, *p* < 0.01; and ***, *p* < 0.001.

In order to study the molecular mechanism of GC cell resistance to OXA, we first established OXA-resistant GC cells (AGS-R and HGC27-R). The GC cells that acquired resistance showed more pronounced OXA resistance with significantly higher IC50 (Figure 5A). Subsequent investigations demonstrated that OXA-resistant cells exhibited a relatively diminished rate of apoptosis upon treatment with OXA (Figure 5B). Furthermore, a noteworthy upregulation in the expression level of METTL3 (Figure 5C) was observed in these resistant cells, accompanied by a concomitant increase in m6A levels (Figure 5D). To further explore the potential role of METTL3 in OXA resistance, we conducted METTL3 knockdown experiments in OXA-resistant cells (Figure 5E). Consequently, we observed a decline in cell viability of OXA-resistant GC cells subsequent to knockdown (Figure 5F). Significantly, upon treating the OXA-resistant cells with OXA after the knockdown of METTL3, a remarkable enhancement in the rate of apoptosis was observed compared to the non-knockdown OXA-resistant GC cells (Figure 5G). The results showed that a decrease in drug resistance occurred in GC cells.

### Combination of METTL3 knockdown and OXA for GC cells provides better results

**FIGURE 6:**
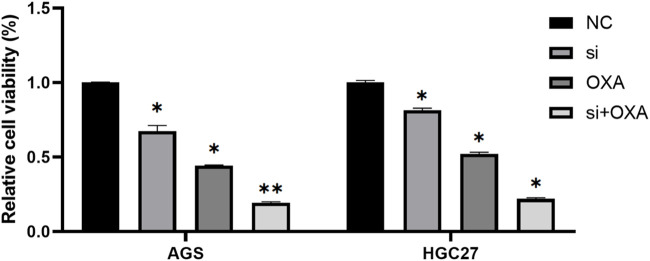
Combination of METTL3 knockdown and OXA for GC cells provides better results. *, *p* < 0.05; **, *p* < 0.01; and ***, *p* < 0.001.

Based on our previous study, it was demonstrated that the targeting of METTL3 could effectively overcome potential resistance to OXA and induce apoptosis. To further explore the potential therapeutic application of targeting METTL3, we compared the cell viability outcomes among the different treatment groups (Figure 6). Our findings revealed that the inhibition of GC cells achieved through the targeting of METTL3 and the use of OXA exhibited an additive effect. Notably, the most substantial inhibition of GC cells was observed when the two treatments were combined.

### DNA repair signaling pathway activates in OXA-resistant GC cells

**FIGURE 7:**
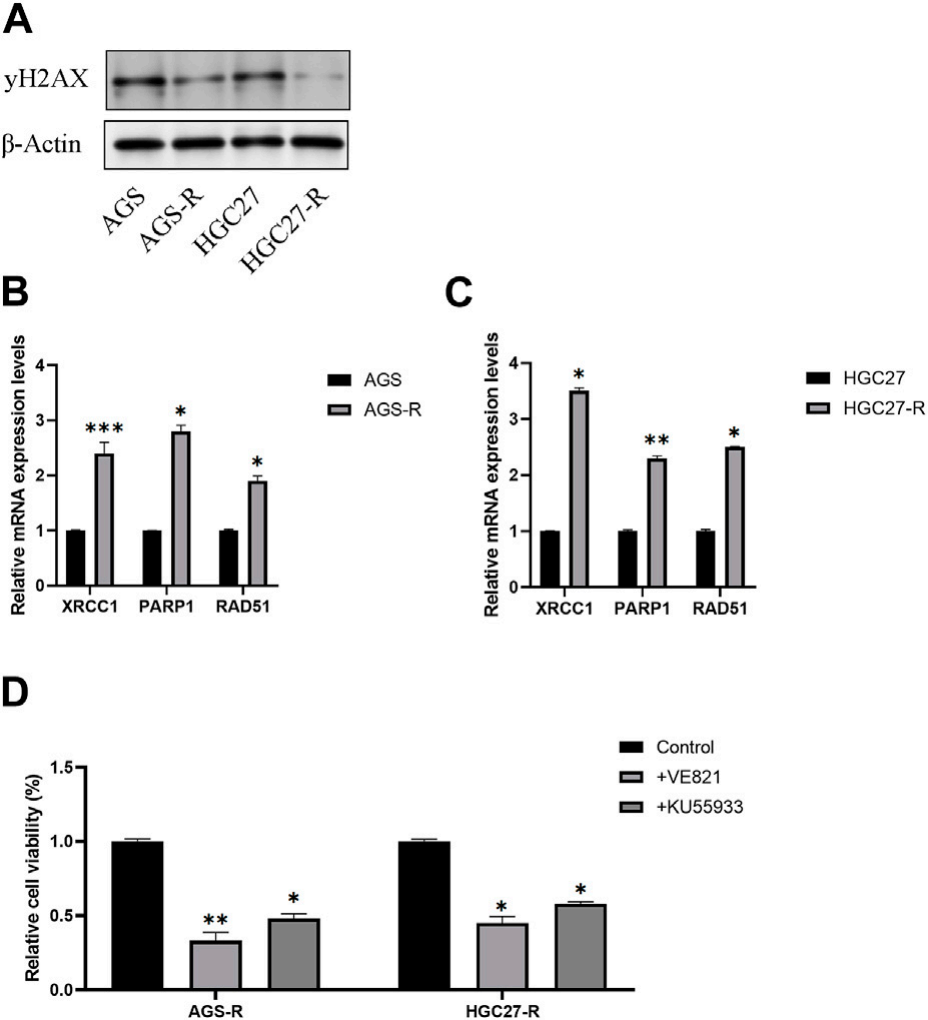
DNA repair signaling pathway activates in OXA-resistant GC cells. **(A)** Protein level examination of γH2AX expression levels in parental GC cells and GC-resistant cells. **(B)** The mRNA expression levels of XRCC1, PARP1, and RAD51 in AGS and AGS-R cells were detected. **(C)** The mRNA expression levels of XRCC1, PARP1, and RAD51 in HGC27 and HGC27-R cells were detected. **(D)** Cell viability of OXA-resistant GC cells was significantly decreased after DNA repair inhibitor treatment. *, *p* < 0.05; **, *p* < 0.01; and ***, *p* < 0.001.

A previous study proposed a potential association between OXA resistance and DNA damage repair mechanisms (Modi et al., 2016). One well-established indicator of DNA damage is the phosphorylated form of H2AX, known as γH2AX. γH2AX plays a crucial role in the DNA damage response by facilitating the recruitment of DNA repair proteins and signaling molecules to the site of DNA damage. Our investigation revealed a noteworthy reduction in the protein levels of γH2AX in OXA-resistant GC cells as compared to the non-resistant parental GC cells (Figure 7A).

Furthermore, we conducted a comprehensive examination of the mRNA expression levels of XRCC1, PARP1, and RAD51, which are key factors in the DNA repair signaling pathway. Our findings demonstrated a significant upregulation of these DNA repair signaling pathway-related factors in GC-resistant cells. Consequently, we concluded that the DNA repair signaling pathway was substantially activated, thus aiding GC-resistant cells in evading apoptosis (Figures 7B, C). To validate our conclusions, we employed DNA repair inhibitors (VE-821and KU-55955). Remarkably, the results indicated a significant reduction in the cell viability of GC-resistant cells subsequent to treatment with DNA repair inhibitors (Figures 7D, E).

### METTL3 regulates OXA-resistance through DNA repair pathway

Our results suggest that OXA resistance in GC cells is associated with DNA repair (Figure 7). Knockdown of METTL3 induces apoptosis in OXA-resistant GC cells. Therefore, we wanted to further investigate whether METTL3 maintains drug resistance in GC cells through DNA repair.

**FIGURE 8:**
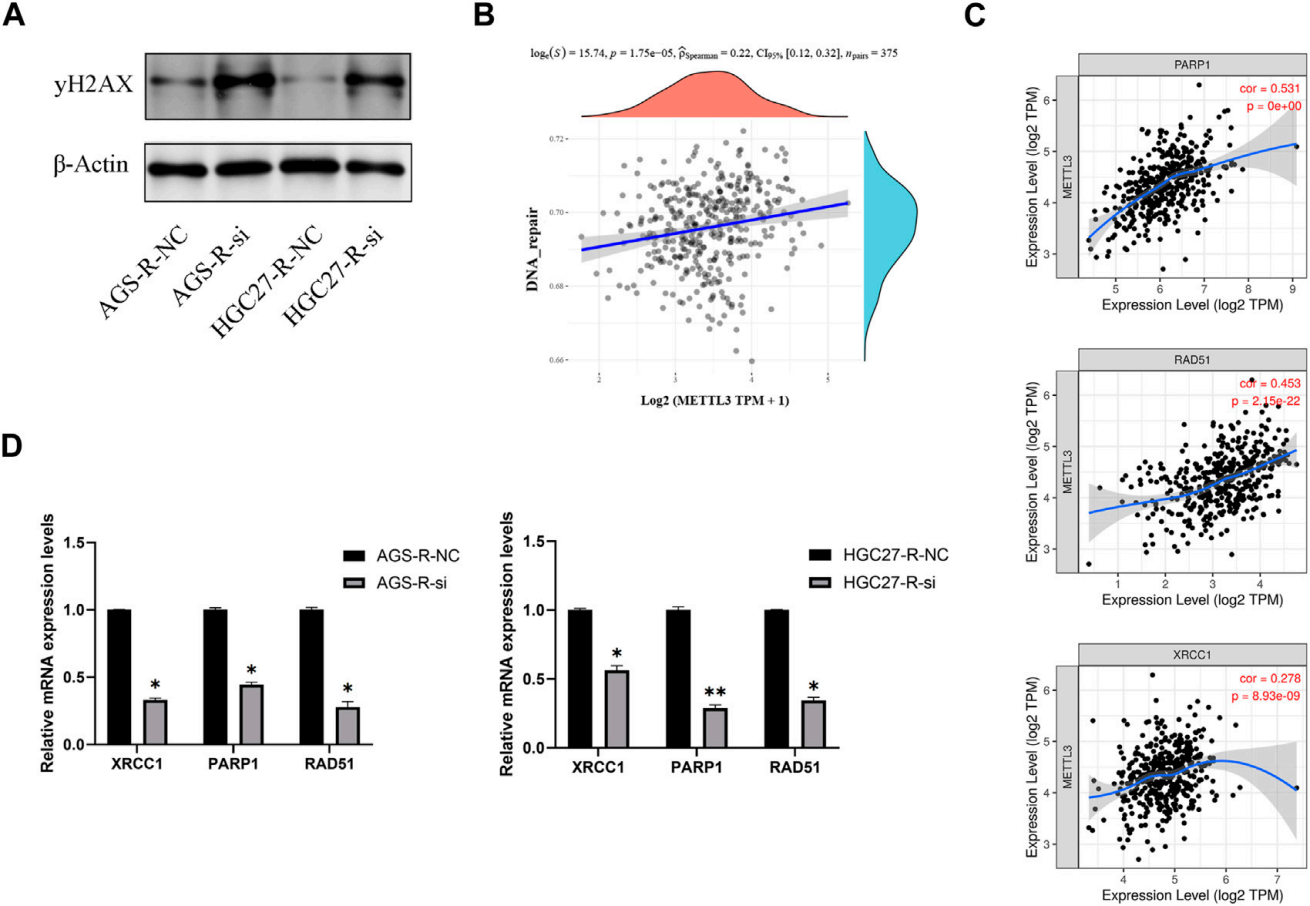
METTL3 regulates OXA-resistance through DNA repair pathway. **(A)** METTL3 knockdown in OXA-resistant GC cells enhances DNA damage. **(B)** A significant positive correlation between METTL3 expression and DNA repair pathways. **(C)** METTL3 was significantly and positively correlated with XRCC1, PARP1 and RAD51. **(D)** DNA repair pathways were significantly inhibited in OXA-resistant GC cells after METTL3 knockdown. *, *p* < 0.05; **, *p* < 0.01; and ***, *p* < 0.001.

To investigate this further, we performed METTL3 knockdown in OXA-resistant GC cells and observed a significant increase in DNA damage after knockdown (Figure 8A). To explore the correlation between METTL3 and DNA repair pathways, we analyzed publicly available data from TCGA-STAD (The Cancer Genome Atlas-Stomach Adenocarcinoma) and found a significant positive correlation between METTL3 expression and the DNA repair pathway (Figure 8B). Specifically, METTL3 showed significant positive correlations with XRCC1, PARP1, and RAD51, which are key players in DNA repair (Figure 8C). RT-qPCR results further validated this analysis by demonstrating significantly reduced mRNA expression levels of XRCC1, PARP1, and RAD51 in OXA-resistant GC cells upon METTL3 knockdown, indicating inhibition of the DNA repair pathway (Figure 8D). These findings suggest that METTL3 may contribute to drug resistance in GC cells by promoting DNA repair. Inhibition of METTL3 could potentially sensitize OXA-resistant GC cells to chemotherapy by impairing their ability to repair DNA damage.

## Discussion

The incidence of gastric cancer has been steadily increasing in recent years, posing a significant challenge in its treatment due to the emergence of drug resistance (Pernot et al., 2015; Riquelme et al., 2016; Deng et al., 2021). Although various chemotherapeutic agents are employed to combat gastric cancer, cancer cells can develop resistance mechanisms that gradually diminish the efficacy of these agents, ultimately leading to treatment failure and disease progression. Among these agents, OXA is commonly used as an adjuvant chemotherapeutic agent for gastric cancer (Qiu et al., 2011; Zhong et al., 2021). Several studies have explored the potential mechanisms of resistance to OXA in gastric cancer, yet the strategies to overcome this resistance are still lacking.

In this study, our focus was on METTL3, a dysregulated gene observed in various cancers and known to be associated with gastric cancer development. Our findings revealed that METTL3 is significantly overexpressed in gastric cancer and is closely linked to poor prognosis. Moreover, the overexpression of METTL3 promotes the proliferation and migration of gastric cancer cells. METTL3 has also been implicated in drug resistance in gastric cancer. For instance, Li et al. demonstrated that METTL3 facilitates PARP1 mRNA stabilization, thereby promoting OXA resistance in CD133+ gastric cancer stem cells by increasing the activity of the base excision repair pathway (Li H. et al., 2022). Additionally, Li et al. discovered that the knockdown of METTL3 enhances 5-FU-induced DNA damage and overcomes 5-FU resistance in HCT-8R cells (Li M. et al., 2022). Consequently, we propose that METTL3 is potentially relevant to OXA resistance in gastric cancer patients, and its knockdown effectively mitigates this resistance. Our results suggested that knockdown of METTL3 induces apoptosis in OXA-resistant GC cells and enhances their sensitivity to OXA. Another important finding is that METTL3 knockdown and OXA treatment exhibited an additive effect of growth inhibition on GC cells. To delve deeper into the mechanisms of gastric cancer drug resistance, we conducted a comprehensive analysis combining bioinformatics and cellular experiments. Our investigation revealed a significant activation of the DNA repair signaling pathway in OXA-resistant gastric cancer cells. Moreover, the inhibition of DNA repair pathways effectively attenuated the resistance of gastric cancer cells to OXA. RT-qPCR analysis further supported the role of METTL3 in regulating OXA resistance through the DNA repair signaling pathway.

DNA repair pathways play a crucial role in maintaining genome integrity by rectifying DNA damage caused by various factors, including chemotherapy drugs (Jeggo et al., 2016; O'Connor, 2015; Baxter et al., 2022). However, the dysregulation of DNA repair mechanisms in cancer cells can contribute to drug resistance. When cancer cells encounter chemotherapy drugs that induce DNA damage, such as platinum-based drugs or radiation therapy, DNA repair pathways can be activated in an attempt to repair the damage (Karran et al., 2003; Asano, 2020; Gao et al., 2021). Unfortunately, this activation can diminish the treatment’s efficacy, enabling cancer cells to survive and proliferate. Efforts to overcome drug resistance mediated by DNA repair pathways are ongoing, necessitating further research to uncover new targets and develop effective therapeutic approaches. Although the involvement of METTL3 in DNA repair is still underexplored, our findings shed light on its significance and its potential in informing the development of novel therapies for gastric cancer.

However, it is important to acknowledge that a major limitation of this study is the lack of validation in animal models and clinical settings. Addressing this limitation and conducting subsequent research in animal models and clinical trials are necessary steps for future investigations.

## Conclusion

Our results showed that METTL3 was highly expressed in OXA-resistant GC cells, and knockdown of METTL3 induced apoptosis in OXA-resistant GC cells. Furthermore, we found that DNA repair inhibitors could reduce OXA resistance. Further studies suggested that METTL3 is involved in regulating drug resistance in GC cells through the DNA repair pathway, and targeting METTL3 combined with OXA therapy could achieve better therapeutic effects.

## Data Availability

The original contributions presented in the study are included in the article/Supplementary Material, further inquiries can be directed to the corresponding author.
