# Supplementary material for: METTL3 promotes drug resistance to oxaliplatin in gastric cancer cells through DNA repair pathway
Source: Front Pharmacol. 2023 Sep 26;14:1257410. doi: 10.3389/fphar.2023.1257410 (PMC10562647; doi:10.3389/fphar.2023.1257410)
Supplement: Supplementary file 1 [file Table1.DOCX]

**Table S1. Sequences of the primers of siRNA.**

| RNAs | Primer sequence (5′ to 3′) |
| --- | --- |
| hs-ME​​TTL3-si-1-F | GUUAGAGAGAGAAGUUGCUAdTdT |
| hs-ME​​TTL3-si-1-R | UAGCAACUUCUUCUCUAACdTdT |
| hs-ME​​TTL3-si-2-F | GGUAAAGCGAGGUCUCCUAdTdT |
| hs-ME​​TTL3-si-2-R | UAGGAGACCUCGCUUUACCdTdT |
| hs-ME​​TTL3-si-NC-F | UUCUCCGAACGUGUCACGUDTDT |
| hs-ME​​TTL3-si-NC-R | ACGUGACACGUUCGGAGAAdTdT |
